# Perirenal Fat in Disease Progression: From Inflammatory Mediator to Therapeutic Target

**DOI:** 10.1111/iju.70158

**Published:** 2025-06-24

**Authors:** Eiji Kashiwagi

**Affiliations:** ^1^ Department of Urology School of Medicine, University of Occupational and Environmental Health Kitakyushu Japan

**Keywords:** adipocytokines, cardiovascular disease, chronic kidney disease, inflammation, metabolic syndrome, perirenal fat

## Abstract

Perirenal fat (PRF), the adipose tissue that encases the kidneys, has long been considered a passive energy reservoir and mechanical buffer. However, emerging evidence has identified PRF as a biologically active visceral fat depot that secretes adipocytokines, inflammatory mediators, and other bioactive molecules, and thereby contributes to the pathogenesis and progression of several systemic diseases. PRF is associated with renal cell carcinoma, cardiovascular disease, chronic kidney disease, obesity, and metabolic syndrome. In renal cell carcinoma, PRF thickness correlates positively with tumor stage and negatively with prognosis. In cardiovascular and renal disorders, PRF accumulation is linked to elevated blood pressure and impaired renal function. Furthermore, PRF promotes a pro‐inflammatory microenvironment that exacerbates chronic kidney disease and metabolic abnormalities. These findings underscore the clinical relevance of PRF, both as a diagnostic and prognostic marker and a potential target for therapeutic intervention. This review provides a comprehensive overview of the roles of PRF in disease development and progression and emphasizes its emerging significance in clinical practice.

## 1 Introduction

**FIGURE 1:**
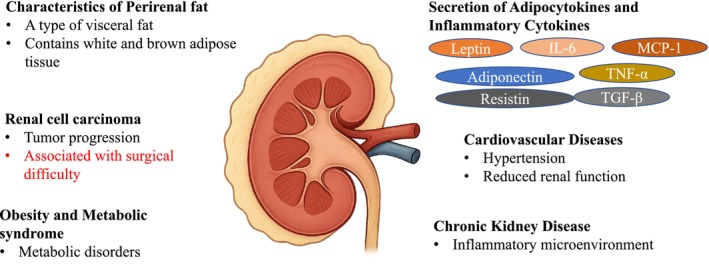
Schematic representation of the multifaceted roles of perirenal fat (PRF), a metabolically active visceral adipose tissue that surrounds the kidney. PRF comprises both white and brown adipose tissue and secretes adipocytokines (e.g., leptin, adiponectin, and resistin) and inflammatory cytokines (e.g., interleukin‐6 [IL‐6], tumor necrosis factor‐α [TNF‐α], transforming growth factor‐β [TGF‐β], and monocyte chemoattractant protein‐1 [MCP‐1]). These bioactive molecules contribute to both local and systemic inflammation and thereby influence the pathogenesis and progression of various diseases. In renal cell carcinoma, PRF is linked to tumor progression and surgical difficulty. In cardiovascular disease, PRF thickness correlates with hypertension and impaired renal function. In chronic kidney disease, PRF contributes to an inflammatory microenvironment and increased intracapsular pressure. Additionally, PRF is implicated in obesity and metabolic syndrome and strongly associated with insulin resistance, dyslipidemia, and subclinical atherosclerosis. These observations underscore the potential of PRF as both a biomarker and therapeutic target across a range of conditions.

Perirenal fat (PRF), the adipose tissue that envelops the kidneys, has historically been regarded as a passive mechanical cushion [1]. However, emerging evidence indicates that PRF is metabolically active: it secretes adipokines and pro‐inflammatory cytokines that influence both renal physiology and systemic metabolic homeostasis [2]. These secretions contribute to the progression of various pathologies, including renal, cardiovascular, and metabolic diseases [2, 3, 4, 5]. Notably, PRF shares functional characteristics with other visceral fat depots, such as pericardial fat, and its excessive accumulation is associated with reduced renal function, hypertension, and chronic kidney disease (CKD) [5]. This review discusses the molecular mechanisms and disease‐specific implications of PRF, with a focus on renal cell carcinoma (RCC), cardiovascular disease (CVD), CKD, and metabolic disorders. By elucidating the complex roles of PRF, this review aims to highlight its clinical relevance and evaluate its potential as a novel therapeutic target (Figure 1).

## 2 Characteristics of Perirenal Fat

Adipose tissue is broadly classified into white adipose tissue (WAT) and brown adipose tissue (BAT) [6]. WAT primarily functions as an energy reservoir, whereas BAT dissipates energy through non‐shivering thermogenesis to generate heat [7, 8, 9]. Although BAT is most abundant in neonates and typically declines with age, recent studies have identified residual BAT activity within adult PRF [10]. Anatomically situated in the retroperitoneal space, PRF constitutes a form of visceral fat [11]. Positron emission tomography imaging has confirmed the presence of metabolically active BAT in the perirenal region, which supports its role in energy expenditure [12, 13].

WAT is the predominant adipose type that surrounds the kidney [5]; it appears as pale yellow, soft, and oily tissue [14]. It mainly consists of large unilocular adipocytes with peripheral nuclei and minimal mitochondrial content. By contrast, BAT appears reddish‐brown, which is due to its high mitochondrial density and rich vascularization. Histologically, BAT is composed of multilocular adipocytes with centrally located nuclei and abundant eosinophilic cytoplasm [14, 15].

In humans, BAT in the perirenal region is typically found in the renal hilum, around the adrenal gland, and along the great vessels [15, 16, 17]. While classical BAT is abundant in infants, adult PRF has been suggested to harbor “beige” adipocytes, inducible brown‐like cells that may arise within WAT depots in response to stimuli such as cold exposure or β‐adrenergic stimulation [18, 19, 20].

Identification of BAT and WAT can be achieved through several approaches. Immunohistochemically, BAT is characterized by strong expression of uncoupling protein 1, a mitochondrial inner membrane protein involved in non‐shivering thermogenesis, whereas WAT lacks uncoupling protein 1 expression [19]. Additional markers of BAT include PR domain containing 16, peroxisome proliferator‐activated receptor‐γ coactivator 1‐α, and cell death inducing DFFA‐like effector A [21, 22, 23], while leptin and resistin are more prominent in WAT [24, 25]. Molecular techniques such as qPCR and Western blotting can further quantify the expression of these markers in dissected fat depots [18, 19].

Although BAT can also be visualized using 18F‐fluorodeoxyglucose positron emission tomography and computed tomography because of its high glucose uptake [12, 13], noninvasive imaging modalities such as magnetic resonance imaging have emerged as useful alternatives. Techniques like proton density fat fraction and Dixon‐based imaging can differentiate BAT from WAT based on differences in water–lipid content and tissue perfusion [26, 27].

Despite the potential for gross visual identification based on tissue color and texture, histological and molecular analyses remain the gold standard for definitive discrimination between BAT and WAT in perirenal adipose tissue.

Cold exposure activates BAT and enhances thermogenic capacity, and therefore represents a potential strategy for managing obesity and type 2 diabetes mellitus (T2DM) [28]. Nevertheless, several challenges hinder the clinical translation of BAT‐targeting therapies. These include the identification of safe and sustainable methods for long‐term BAT activation, interindividual variability in BAT responsiveness, and the risk of adverse effects such as increased cardiovascular stress [29]. Additionally, the feasibility and cost‐effectiveness of large‐scale implementation remain significant barriers.

Importantly, WAT can undergo transdifferentiation into beige adipose tissue, which exhibits thermogenic properties akin to BAT [30]. Pharmacological approaches aimed at promoting this browning process are currently under investigation and may offer promising therapeutic avenues for metabolic disorders [31].

## 3 Adipocytokines and Inflammatory Cytokines

As a metabolically active visceral fat depot with vascular and neural connections, PRF secretes a variety of bioactive substances, including adipocytokines and inflammatory cytokines [2, 4, 32, 33, 34]. These secretions contribute to both local and systemic inflammatory responses and link PRF to the pathogenesis of CKD, CVD, and metabolic disorders [2, 34]. Key adipocytokines such as leptin and adiponectin, along with pro‐inflammatory cytokines including interleukin‐6 (IL‐6) and tumor necrosis factor‐α (TNF‐α), are secreted by PRF and play pivotal roles in metabolic dysregulation and disease progression.

Understanding the mechanisms through which PRF mediates these effects is essential, as it opens avenues for novel therapeutic interventions. Targeting adipocytokine signaling and inflammatory pathways in PRF may help mitigate renal and metabolic dysfunction [35]. Although further investigation is warranted, these insights support the growing recognition of PRF as an active contributor to disease processes rather than a passive fat depot.

In particular, leptin secreted from PRF has been implicated in the development of renal fibrosis and glomerulosclerosis. Experimental studies have shown that leptin promotes the expression of profibrotic mediators such as transforming growth factor‐β (TGF‐β) and collagen type I in renal cells, including glomerular endothelial and mesangial cells; this may occur via signaling pathways such as JAK/STAT and mitogen‐activated protein kinase (MAPK), although the precise mechanisms remain to be fully elucidated [36, 37]. These molecular alterations, particularly the increased production of pro‐inflammatory cytokines such as TGF‐β, contribute to extracellular matrix deposition, tissue remodeling, and interstitial fibrosis, and therefore link PRF‐derived adipocytokines to the pathogenesis and progression of CKD [38, 39]. Additionally, leptin promotes sympathetic nervous system activity, endothelial dysfunction, and sodium retention, all of which exacerbate hypertension and further impair renal function [40, 41].

Adiponectin, another major adipocytokine secreted by adipose tissue, generally exerts anti‐inflammatory and insulin‐sensitizing effects [42]. However, its expression is markedly reduced in dysfunctional or inflamed PRF, particularly in obesity and diabetes [43]. This reduction in adiponectin is associated with increased renal oxidative stress, macrophage infiltration, and podocyte apoptosis, which further links adipose dysfunction to renal pathophysiology [44, 45, 46]. Notably, experimental models have demonstrated that exogenous adiponectin administration can attenuate albuminuria and glomerular injury, which suggests that this adipokine has a protective role that is lost in PRF‐mediated disease states [47]. Secretion of the adipocytokines TNF‐α, IL‐6, and monocyte chemoattractant protein‐1 by PRF also contributes to local inflammation and systemic metabolic disturbances [48]. Among them, TNF‐α is a potent activator of both the nuclear factor‐κ B and MAPK signaling pathways [49]; IL‐6 also activates MAPK signaling and, to a lesser extent, nuclear factor‐κ B signaling pathways [50]. Monocyte chemoattractant protein‐1, through its receptor C‐C motif chemokine receptor type 2, recruits and activates monocytes and macrophages, and therefore also contributes to the activation of these signaling pathways, particularly in immune cells [51]. Activation of these signaling cascades in renal parenchymal cells promotes the production of additional cytokines, reactive oxygen species, and fibrotic mediators, and further exacerbates renal inflammation and fibrosis [52, 53]. Histologically, PRF from individuals with obesity or CKD often displays increased infiltration of macrophages and elevated expression of inflammatory genes [54].

In summary, PRF represents a dynamic and metabolically active tissue that contributes to the modulation of renal, cardiovascular, and metabolic homeostasis through its secretion of adipocytokines and inflammatory mediators. Understanding these complex interactions may yield novel strategies for the prevention and treatment of PRF‐related pathologies.

## 4 Renal Cell Carcinoma

The role of PRF in RCC is multifaceted and, at times, paradoxical. Obesity is a well‐established risk factor for RCC, particularly for the clear cell subtype (ccRCC) [55]. Interestingly, several studies have reported that increased PRF volume and lower fat radiodensity are associated with improved progression‐free and overall survival in patients with ccRCC [56, 57]. This phenomenon supports the so‐called obesity paradox, wherein greater fat accumulation appears to confer a protective effect against disease progression [57].

However, findings are not universally consistent. Other studies have demonstrated that increased PRF thickness correlates with poorer progression‐free survival in patients with localized ccRCC [56]. The Mayo Adhesive Probability (MAP) score—an imaging‐based metric assessing the quality of perinephric fat—has emerged as a predictor of surgical difficulty during nephrectomy [58]. High MAP scores, increased PRF thickness, and pronounced perinephric stranding are collectively associated with “toxic fat,” which complicates tumor dissection and increases the risk of intraoperative complications [56].

Although brown adipocytes have been identified within PRF depots [59], the relationship between “toxic fat” and BAT remains poorly understood. Toxic fat, as observed in cases with a high MAP score, is characterized by fibrosis, immune cell infiltration, and a pro‐inflammatory cytokine milieu [60, 61]. However, these pathological features have not been clearly associated with either the activation or suppression of BAT. It remains unclear whether the development of toxic fat represents a reduction in BAT‐like features or reflects an entirely distinct pathological process. Further investigation is needed to clarify whether the fibrotic transformation of perirenal adipose tissue is associated with impaired thermogenic capacity or browning potential.

Although a direct link between the MAP score and systemic inflammation has not been established, evidence suggests an indirect association. The MAP score correlates with the presence of adhesional perinephric fat, which is implicated in systemic inflammatory responses. The development of adhesional perinephric fat appears to involve the activation of chronic inflammatory pathways, including the upregulation of pro‐inflammatory cytokines such as IL‐6 and TNF‐α [58]. Notably, the MAP score has also been identified as a predictor of progression‐free survival in patients with localized RCC [62].

Recent evidence suggests that ccRCC interacts dynamically with the surrounding PRF to contribute to a tumor‐promoting microenvironment. In a study using both human ccRCC samples and murine models, Wei et al. demonstrated that tumor‐derived parathyroid hormone‐related protein may promote browning‐like features in adjacent perinephric adipose tissue via activation of the protein kinase A signaling pathway, and ultimately lead to increased thermogenic activity that fuels tumor progression [63].

Conversely, PRF actively contributes to tumor progression in ccRCC through the secretion of bioactive molecules [34]. Secretion of the adipokines leptin, adiponectin, and resistin, along with pro‐inflammatory cytokines including IL‐6 and TNF‐α [34, 42], by PRF can promote tumor cell proliferation, angiogenesis, and immune evasion. Moreover, PRF influences the tumor microenvironment by modulating immune cell infiltration and cytokine profiles, and thereby shapes a tumor‐permissive immune landscape in RCC [48, 64, 65].

Thick PRF in RCC is associated with a higher risk of outward tumor protrusion, potentially due to the lower pressure environment within PRF compared with the renal parenchyma [66]. Recent findings from transcriptomic analysis of tumor and PRF tissues, along with in vitro studies, have further elucidated the role of PRF in the obesity paradox observed in renal cancer [57]. Overall, the interplay between PRF and RCC is multifaceted, involving metabolic, anatomical, and inflammatory components that influence tumor progression and clinical outcomes.

## 5 Cardiovascular Diseases

PRF thickness has emerged as an independent risk factor for CVD. Elevated PRF thickness is significantly associated with increased incidence of hypertension, insulin resistance, and dyslipidemia [67, 68]. Mechanistically, enlarged PRF pads elevate intracapsular pressure, compress renal vasculature, and secrete proatherogenic cytokines, thereby promoting atherosclerosis and contributing to cardiorenal dysfunction [69, 70]. Owing to its modifiable nature and quantifiability via imaging, PRF thickness holds promise as both a diagnostic marker and therapeutic target in CVD prevention and management strategies [71].

## 6 Chronic Kidney Disease

In patients with T2DM, PRF thickness is a key predictor of CKD progression. Increased PRF volume can compress renal vasculature, which leads to elevated intracapsular pressure and the development of congestive nephropathy [70]. Comparative studies have shown that PRF is a stronger determinant of CKD risk than total body fat or subcutaneous fat [72]. These findings highlight the potential of PRF as a target for therapeutic intervention in CKD, particularly among individuals with obesity or diabetes [73]. Interventions may include lifestyle modifications, such as tailored dietary and exercise programs, as well as pharmacological treatments aimed at modulating adipocytokines and inflammatory responses. Moreover, advances in imaging techniques may facilitate real‐time monitoring of PRF changes, which would enable individualized treatment approaches.

## 7 Obesity and Metabolic Syndrome

PRF thickness is closely linked to metabolic disorders, including hypertension, diabetes, and dyslipidemia [68]. It correlates strongly with anthropometric and biochemical markers such as body mass index (BMI), waist circumference, and glycated hemoglobin levels [74]. Compared with other adipose tissue compartments, PRF has a superior predictive value for subclinical carotid atherosclerosis in patients with T2DM [75]. Its unique anatomical position and endocrine activity underscore its pivotal role in the pathophysiology of metabolic syndrome, which further supports its potential as a therapeutic target.

In addition to its associations with metabolic disorders, PRF thickness correlates significantly with various anthropometric indicators of obesity. Studies have reported moderate to strong positive correlations between PRF thickness and BMI, waist circumference, and subcutaneous fat thickness [75]. For example, PRF thickness is an independent predictor of metabolic syndrome in patients with steatotic liver disease, even after adjusting for BMI and waist circumference [68]. Similarly, PRF thickness outperforms BMI and other conventional obesity markers in predicting subclinical carotid atherosclerosis in patients with T2DM [76]. These findings underscore the utility of PRF as a unique and potentially superior biomarker of visceral adiposity and cardiometabolic risk, and highlight its relevance beyond traditional measures such as BMI or waist circumference.

## 8 Strategies for Modulating PRF

β3‐adrenergic receptor agonists were originally developed as anti‐obesity agents, based on their ability to activate BAT and promote thermogenesis in animal models. However, early clinical trials in humans were largely unsuccessful because of limited β3‐receptor expression and poor efficacy [77]. Interestingly, one such agent, mirabegron, was repurposed and later approved for the treatment of overactive bladder. In recent years, mirabegron has been shown to activate BAT in humans, enhance energy expenditure, and improve insulin sensitivity, which suggests a potential role for this drug in the management of metabolic disorders [78, 79]. Although not originally intended as a therapeutic strategy for obesity or diabetes, β3‐agonists now represent a promising pharmacological approach to modulate PRF activity and systemic metabolic function. Further clinical trials are warranted to clarify their long‐term safety and disease‐specific benefits.

Recent studies have demonstrated that combined therapy with metformin and dapagliflozin significantly reduces PRF thickness, which improves insulin sensitivity and reduces inflammatory markers [80]. Additionally, novel agents targeting inflammatory pathways, such as NOD‐, LRR‐, and pyrin domain‐containing protein 3 inflammasome inhibitors, have shown potential in reducing adipose tissue inflammation and metabolic complications, although their direct effects on PRF remain to be elucidated [81].

There are very few clinical studies or reviews focusing specifically on the surgical removal of PRF in humans. While some animal studies have investigated the physiological effects of PRF excision [82], such approaches have not been applied as standard clinical practice or therapeutic interventions in humans. In RCC surgery, PRF is typically removed en bloc with the kidney during radical nephrectomy, whereas only a portion of the PRF is excised during partial nephrectomy. However, the clinical significance and impact of selective PRF excision alone remain unclear and represent an important area for future research.

## 9 Conclusion

PRF is no longer viewed as an inert structural component but rather as a dynamic and metabolically active tissue that exerts far‐reaching effects on renal, cardiovascular, and metabolic physiology. PRF is a type of visceral fat, and like other visceral adipose depots, it secretes adipocytokines and pro‐inflammatory cytokines. However, its anatomical proximity to the kidneys means that PRF has a particularly strong local impact on renal function and blood pressure regulation. Its rich vascularization and neural connections, particularly in BAT‐rich regions, along with its ability to secrete a broad array of adipokines and cytokines, underscore its unique role in both local and systemic disease processes.

Accumulating evidence implicates PRF in the pathogenesis and progression of multiple diseases, including CKD, hypertension, T2DM, and RCC. Notably, PRF contributes to disease through both mechanical mechanisms, such as increased intracapsular pressure and parenchymal compression, and molecular signaling pathways involving inflammation, fibrosis, and immune modulation. Moreover, PRF exhibits histological and functional heterogeneity, with features that may reflect both white and brown adipose phenotypes, though the clinical relevance of this plasticity remains to be fully elucidated.

From a clinical perspective, PRF thickness has emerged as a noninvasive imaging biomarker that correlates with metabolic risk factors and predicts adverse cardiovascular and renal outcomes more effectively than traditional anthropometric indices. These observations suggest that PRF assessment may hold diagnostic, prognostic, and therapeutic value in the management of cardiometabolic and renal disorders.

Future research should focus on elucidating the temporal and causal relationships between PRF remodeling and disease progression. Unanswered questions include whether interventions that modulate PRF volume or its secretory profile, such as pharmacologic agents, lifestyle changes, or bariatric procedures, can directly improve renal or cardiovascular outcomes.

In summary, PRF is an emerging and clinically relevant target at the intersection of metabolism, inflammation, and organ‐specific pathology. Elucidating its pathophysiological roles and therapeutic potential may provide new strategies for combating complex, multifactorial diseases such as RCC, CVD, CKD, and metabolic syndrome.

## Author Contributions

The author performed all tasks related to this study and manuscript.

## Ethics Statement

The author has nothing to report.

## Conflicts of Interest

The author declares no conflicts of interest.

## Data Availability

This article is a review and does not include any original data. Therefore, data sharing is not applicable.
